# Risk Factors for Amputation in the Surgical Treatment of Hemophilic Osteoarthropathy: A 20-Year Single-Center Report

**DOI:** 10.1155/2022/1512616

**Published:** 2022-03-28

**Authors:** Yiming Xu, Bin Feng, Wei Zhu, Yingjie Wang, Xisheng Weng

**Affiliations:** ^1^Department of Orthopedics, Peking Union Medical College Hospital, Chinese Academy of Medical Sciences & Peking Union Medical College, Beijing 100730, China; ^2^State Key Laboratory of Complex Severe and Rare Diseases, Peking Union Medical College Hospital, Chinese Academy of Medical Sciences & Peking Union Medical College, Beijing 100730, China

## Abstract

**Purpose:**

Hemophilic osteoarthropathy (HO) is a common cause of spontaneous bleeding in hemophiliacs. Surgical procedures are necessary for patients with severe HO. As a last resort, amputation is sometimes needed to treat complex HO cases. This study aimed to review the existing records of patients who underwent amputations in HO surgical treatment, summarize the risk factors, and provide relevant references for surgeons.

**Methods:**

We retrospectively reviewed the records of hemophilic patients received surgeries at Peking Union Medical College Hospital between 2000 and 2020. The amputation patients without neoplasm or acute trauma were screened out. Patient information was extracted from medical records. A literature retrieval of hemophilic amputation cases was conducted via PubMed. The risk factors of amputation were summarized and analyzed via descriptive statistics and Fisher's precision probability test.

**Results:**

Four male hemophilia A patients out of 201 patients underwent lower limb amputation. The reasons of amputation contained severe pseudotumor with factor inhibitor and large bone defects, recurrent pathological fracture with pseudotumor, skin ulcer with chronic osteomyelitis, and pseudotumor with systematic infection. In cases reported in retrieved literature, severe pseudotumor with complications, bacterial infection and factor inhibitor were common factors. *Discussion*. As the first study on amputations in HO patients, we found that severe hemophilic pseudotumor, chronic bacterial infection, and coagulation factor inhibitor was potential risk factors for amputation. Sufficient factor replacement therapy is fundamental in the prevention of amputation. The early diagnosis and specially designed surgical techniques could improve the rate of limb salvage.

## 1. Introduction

Hemophilia comprises hereditary hemorrhagic disorders characterized by coagulation system deficiencies. The most common cause of hemophilia is coagulation factor VIII or IX deficiency, causing hemophilia A or B, respectively [1]. Coagulation factor deficiencies lead to spontaneous intra-articular or intramuscular bleeding. The persisting chronic stimulation of blood stasis causes musculoskeletal lesions called hemophilic osteopathic medicine (HO). Recurrent hemarthroses consequent to bleedings contributed to cartilage degeneration named hemophilic arthritis. [2]. Inappropriate treatment of intramuscular bleeding may cause soft tissue hematoma called hemophilic pseudotumor [3]. Hemophiliacs also have a higher prevalence of osteoporosis and pathological fractures [4, 5]. HO is associated with serious pain and motion dysfunction, especially in hemophiliacs administrated with limited hemostatic agents during childhood, and required surgical interventions for pain relief [6, 7].

With the developments of coagulation factor replacement therapy and perioperative management, hemophiliacs can receive safe surgical procedures. Total joint arthroplasty is used to treat end-stage hemophilic arthritis [8]. Surgical resection is applied in treating hemophilic pseudotumor [9]. In the early years, amputation of extremities was used in severe HO cases due to imperfect surgical technology [10]. Unfortunately, there are still amputation cases reported in modern literature, with no systematic summary for amputation risk factors and prevention strategies [9, 11, 12]. In this study, we retrospectively evaluated amputation cases in the surgical treatment of HO, summarizing their clinical features and risk factors. As the first study on amputations in hemophilia patients, we hope that the result can provide a reference for surgeons facing the hard choice of limb salvage or amputation.

## 2. Materials and Methods

We retrospectively reviewed the medical records of hemophiliacs who underwent surgery for HO at Peking Union Medical College Hospital between 2000 and 2020. This study was approved by the appropriate institutional ethics review board. The patients who received amputation of extremity or joint disarticulation were screened out. Patients were excluded if the amputation was performed due to nonhemophilic diseases such as neoplasm or acute trauma. We recorded the following data: demographic data, type and severity of hemophilia, presence of factor inhibitor, aetiological antecedent, clinical preferences, and management. For all included amputation patients, follow-ups were performed by telephone, Internet, or interviews.

Because amputation is rare in hemophiliacs, we conducted literature retrieval using PubMed. The search terms contained “hemophilia,” “amputation,” and “disarticulation”. The publication type was not limited. Clinical characteristics and amputation reasons of all reported cases were also recorded.

The results about amputation cases were shown in descriptive statistics and Fisher's precision probability test. Summary statistics were performed using Microsoft Excel 2016 (Microsoft, Redmond, WA) and SPSS Statistics 19.0 (SPSS Inc, Chicago, IL, US). Numerical data were summarized in tables as arithmetic means ± standard deviation. Nominal and ordinal data are shown as frequencies with percentages. The level of significance was defined at *p*=0.05.

## 3. Results

### 3.1. General Characteristics

In total, 201 patients with 262 surgical procedures were included. Four patients underwent four amputation surgeries, with a prevalence of 1.99% (4/201). None of the patients suffered neoplasm or acute trauma, so no case was excluded (Table 1). All four amputation patients were male with hemophilia A: two of them had received regular factor replacement before and two only received factor replacement on demand. Factor inhibitor was found in one patient. Only one case directly received amputation rather than as a secondary procedure. From the literature, we identified 27 amputation cases from 14 papers published between 1992 and 2021. The majority of reported amputation cases had hemophilia A (23/27, 85.19%). Inhibitors were identified in seven hemophilia A cases and one hemophilia B case (Table 2).

### 3.2. Reasons for Amputation

**Figure 1:**
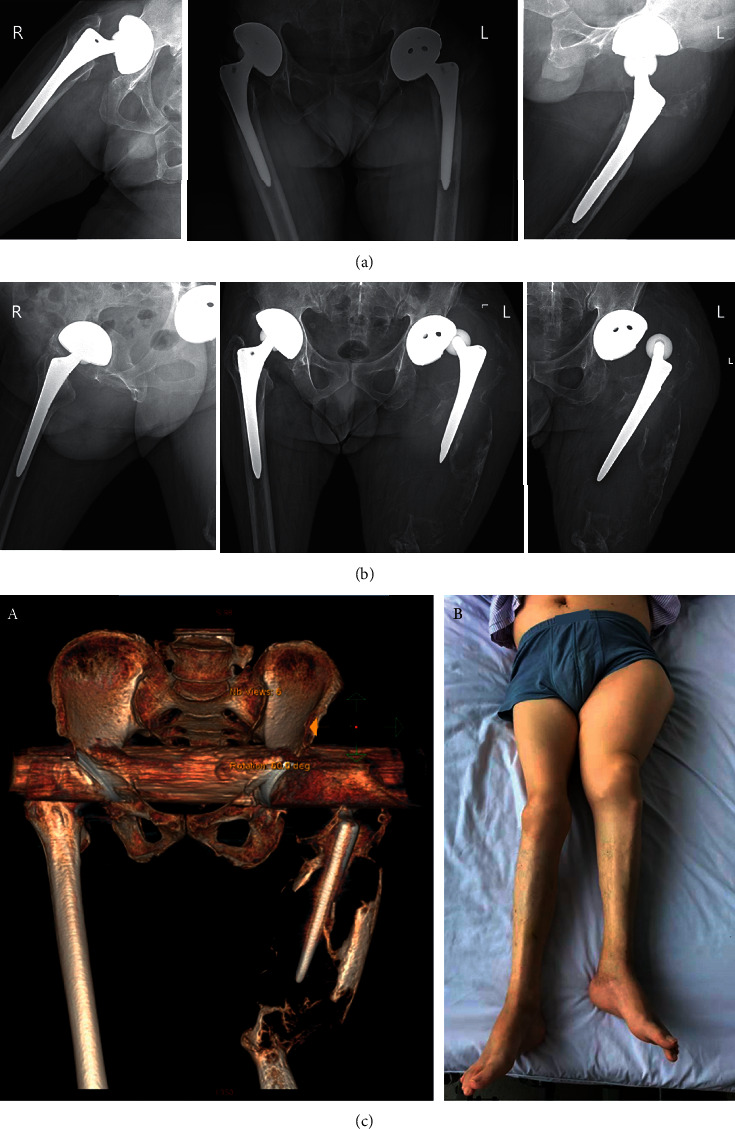
X-ray photography of hip joints of Patient 1. (a) Taken two months after trauma. (b) Taken eight months after trauma, nearly the entire left femur displayed a characteristic “soap bubble” appearance, and the femoral stem “floated” in a large pseudotumor. (c) CT reconstruction (A) and general photography (B) taken eight months after trauma.

**Figure 2:**
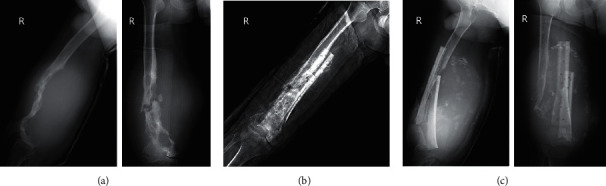
X-ray photography on the right femur of Patient 2. (a) The recurrent hemophilia pseudotumor before the second procedure (internal fixation with an allogeneic bone graft). (b) The postoperative situation of fracture fixation. (c) The collapse of internal fixation and recurrent pseudotumor two years after internal fixation.

Reasons for amputation varied among patients. The reasons for four amputations were variable (Table 2). Patient 1 accidently broke his left hip five years after bilateral total hip arthroplasty (THA). In the local hospital, he was diagnosed with a left hip periprosthetic fracture, received factor VIII infusion with limb braking for two months. The fracture did not heal, and factor inhibitor (6.8 BU/ml) was the first time found for him. After six months' termination of factor replacement, the activity of the inhibitor decreased to 1.9 BU/ml, but the left thigh swelled significantly, which was a large pseudotumor with severe bone defects in radiograph (Figure 1). No conventional surgical technique except amputation could fix it. Patient 2 suffered a pathological fracture of the right femur caused by trauma. He received five months' bone traction at a local hospital. Two years later, a pseudotumor with bone defects was identified in the right inner thigh. In the next four years, he received twice resection of pseudotumor and one open reduction with allograft bone graft fixation of pathological fracture in the defected site. However, the fixation failed with the recurrence of pseudotumor. Methicillin-resistant *Staphylococcus epidermidis* was identified within wound secretion. The fracture, pseudotumor, and infection led to amputation (Figure 2(c)).

**Figure 3:**
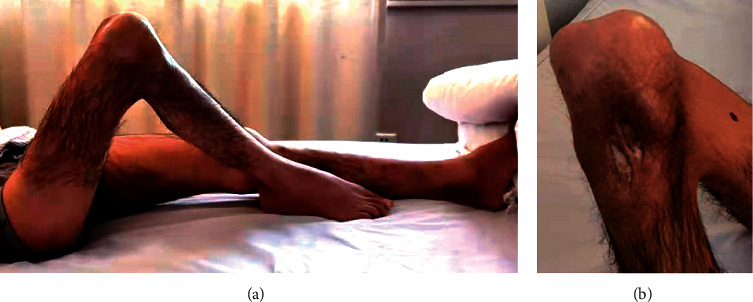
(a) Lateral photography of Patient 3 in supine position before amputation. (b) Partial photography of posterior side of Patient 3's right knee.

**Figure 4:**
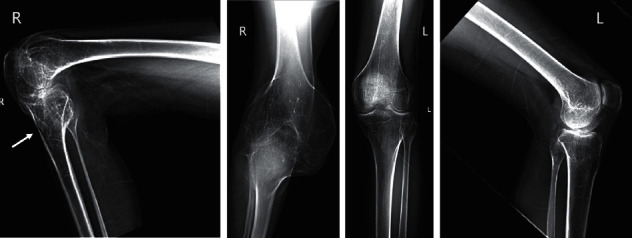
X-ray photography on both knees of Patient 3 before amputation.

Patients 3 and 4 lost walking ability for years because of HO in knees. Patient 3 received regular factor replacement and developed two 3 cm ulcerative wounds on the right proximal tibia in the third year on the hospital bed. A surgical debridement attempt failed. Twelve years later, his right knee was fixed at a 90 angle (Figure 3). X-ray findings suggested a sinus between the wound and tibia bone marrow, indicating suspected chronic osteomyelitis (Figure 4). A similar 1 cm abrasion was found on the right inner thigh of Patient 4 after minor seizures for six years on the hospital bed. Two years later, the whole right lower limb began swelling and bleeding. Cultivation of wound secretions revealed the presence of *Pseudomonas aeruginosa*. Systematic application of antibiotics failed to control the persistent fever (37.5°C). Chronic infections were responsible for their amputations.

Among these four patients, the most common reasons for amputation were pseudotumor (3/4, 75%) and bacterial infection (3/4, 75%). The statistical analysis showed a significant difference of these factors between amputation and nonamputation cases (*p*=0.001). In cases reported in literature, hemophilic pseudotumor (24/27, 88.89%) and bacterial infection (14/27, 51.85%) were also common factors. There was only one amputation patient carried factor inhibitor in our four patients. However, he was the only inhibitor carrier among included patients. The previous amputation cases contained seven cases carrying inhibitor (25.93%), indicating that inhibitor could be another potential risk factor (Table 2).

### 3.3. Perioperative Treatment and Follow-Up

**Figure 5:**
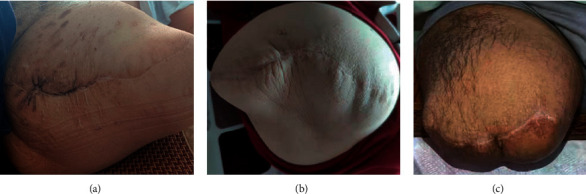
The healing situation of patients in the latest follow-up. (a) Patient 1 in 3 years after amputation. (b) Patient 2 in 3 years after amputation. (c) Patient 3 in 1 year after amputation.

All amputation patients underwent coagulation factor replacement during perioperative period under the guidance of hematologists. Two patients received aboveknee amputation while hip disarticulation and high-thigh amputation were separately performed on the other two patients. The last follow-up assessment was conducted in June 2021 (Table S1). Patient 1 was the only patient suffered from severe postoperative complications. He used recombinant human factor VIIa (rhFVIIa) and human prothrombin complex concentrate (PCC) for perioperative replacement. The FVIII: I level was beyond the limit (>64 BU/ml) with progressive anemia (96 to 43 g/l) and increased wound drainage (40 to 230 ml/d); thus, factor replacement was halted. In the next 63 days, he underwent three surgical debridement under intermittent factor replacement. Consequently, the inhibitor level decreased to 35.2 BU/ml. *Staphylococcus epidermidis* was once detected in wound secretions, so vancomycin was systematically applied. He was transferred to a local hospital 68 days after amputation, and the incision healed completely after another 40 days of regular dressing. Patient 4 was transferred to the intensive care unit after surgery for three days due to a systematic infection. He recovered well and was discharged after the removal of stitches. Two years later, he died from a major epileptic event, which was found before the abrasion of the knee. In the last follow-up, all remaining patients reported no complaints, partly recovered self-care ability with crutches (Figure 5).

## 4. Discussion

At present, amputations are mainly used in chronic nonhealing ulcers or severe infections [13, 14]. This paralytic operation reduces life quality, so limb salvage is usually the first choice [15]. Though significant progress has been recorded in surgical techniques for hemophiliacs, amputations are still the final option for some severe HO cases. However, to our knowledge, no report has specially investigated the reasons for amputation in hemophiliacs. Due to the low incidence, these amputations have only been reported in isolated case reports or studies with different aims (Table 3). The present study described four amputation cases of hemophilia patients who underwent surgical treatment in a single medical center with an average follow-up of 2 years.

Severe hemophilic pseudotumor was seen in three of our four cases, which is also common in previously reported amputation cases. Recurrent spontaneous bleeding promotes the enlargement of the hematoma, resulting in incredible limb swelling and disability. The circumference of the lower limb could be several times larger than the normal value (Figure 1(c)). In extreme cases, pseudotumor compression can cause severe compartmental syndrome [21]. Pathological fracture or osteolysis is seen in half of pseudotumor amputations (2/4 in the present study and 12/24 in previous reports). The fracture is often the initial pseudotumor cause or complication. Osteoporosis is a potential explanation for these fractures. The prevalence of bone mineral density (BMD) reduction can be up to 50%–80% in hemophiliacs [4]. Systematic analyses have suggested a relationship between low BMD and severe hemophilia, but precise mechanisms linking the conditions remain unclear [28, 29]. Osteolysis is mainly seen in patients with massive or multiple pseudotumor. The loss of BMD is accompanied by shape deformation. In X-ray imaging, the affected bone has a characteristic bubble-like shape and the bone will finally disappear in the pseudotumor. The exact mechanism of the osteolysis remains unclear; inflammatory reactions during hematoma absorption may induce bone resorption. As a result of fracture and osteolysis, there is a high risk of recurrent internal/external fixation failure and fracture nonunion, leading to amputation. For patients with huge osteolytic defects, total femur replacement or custom-made prosthesis is applied in some limb salvage cases, but the long-term outcomes require further observation [30, 31]. Surgeons should be aware of unexplained swelling of extremities in hemophiliacs, especially the patients with a history of trauma or poor factor replacement. Adequate factor replacement is the first step in preventing bleeding and controlling pseudotumor. Based on the experience of Patient 1 in our study, we suggest regular radiology checks for osteolysis spaced at a time interval of no more than six months for patients with pseudotumor. The earlier discovery of osteolysis means a higher possibility of limb salvage. Perioperative and postoperative factor replacements are key factors affecting the success of surgical procedures in hemophiliacs; bleeding owing to inadequate replacement worsens the healing situation, and the acute hematoma may cause thrombosis via compression [27].

In the present study, the relationship between infection and amputation was highlighted. Abrasions or small wounds are common on swollen limbs with pseudotumor. Coagulation disorders make curating these wounds very hard, leaving them at high risk of chronic bacterial infection. The surgical removal of infection foci is a must for these patients. Control of coagulation disorder is the fundamental infection prevention solution. The replacement of infected joints is also a high-risk factor for amputation. A retrospective survey of the Danish population identified prosthetic joint infections as the most common cause of limb amputation after primary total knee arthroplasty [32]. Active cutaneous or deep tissue infections are important risk factors for prosthetic infections [33]. There are also reports of amputations in hemophiliacs caused by uncontrolled infection after knee arthroplasty [22]. So, patients with chronic infection or unhealed wounds are not suitable for joint arthroplasty. Arthrodesis may be a safer choice to avoid amputation.

In all hemophiliacs included in the current study, Patient 1 was the only inhibitor carrier. Meanwhile, about a quarter of reported amputation cases had inhibitors, most of which underwent amputation as the first surgical treatment. It seems that hemophiliacs with inhibitors had higher possibility of amputation. The existence of inhibitors, antifactor antibodies, decreased the activity of coagulation factors, making hemostasis management difficult [34]. The etiology of developing factor inhibitors remains incompletely understood. Current evidence suggests a potential relationship between environmental factors (surgery, severe bleeds, and infections) and factor inhibitors [35]. Bypass agents such as rhFVIIa and PCC have adequate hemostatic effects and low rates of complications in surgical procedures involving inhibitor-positive patients [36–38]. Sufficient factor replacement plays a more important role in the perioperative management of hemophiliacs with factor inhibitors. Patient 1 in the present study was switched from rhFVIIa to PCC only three days after surgery for economic reasons. Then, he suffered from bleeding and incision complications. We advise that factor replacement via bypass agents should be extended.

There are some other risk factors for amputation. Most amputation patients are diagnosed with moderate or severe hemophilia A, indicating the influence of hemophilia type and severity. However, the prevalence of hemophilia A is about six times higher than that of hemophilia B, which may affect the incidence of severe HO and amputation. Socioeconomic factors also influence the amputation rate [1]. As we discussed above, sufficient factor replacement is important to prevent amputation. It is reported that 51% of people in high- and upper-middle-income countries use 94% and 92% of the total global quantity of factors VIII and IX, respectively [39]. For patients in developing countries, like Patient 2 in our study, regular replacement is still too expensive. Megaprothesis for osteolysis is also costly; patient 1 in our study could not afford this. In the future, gene therapy might completely eradicate this hereditary disease, but economic issues will still be barriers for many patients [40].

The retrospective nature of this study is a limitation. Furthermore, this study involved a heterogeneous group, a small number of cases, and lacks a randomized control group. Considering that amputation is rare in hemophilia treatment, a multicenter review may provide additional evidence and enhance the meaningfulness of the result.

In general, amputation of the extremities is the last but necessary option for patients with severe HO. Severe hemophilic pseudotumor, chronic bacterial infection, and factor inhibitors are threatening risk factors for amputation in hemophiliacs. Sufficient factor replacement therapy is fundamental in the prevention of amputation. Early diagnosis of severe complications such as osteolysis and special surgical techniques such as total femur replacement could be useful in limb salvage.

## Figures and Tables

**Table 1:** Patient characteristics, management, and outcome of amputation cases.

Patient	Type and severity	Inhibitor	Duration until amputation	Daily factor replacement	Initial diagnosis of HO	Management before amputation	Direct reason for amputation	Amputation procedure	Outcome
1	A, severe	Yes, active (6.8 ⟶ 1.9 BU/ml)	21 years	Regular replacement as secondary prophylaxis (FVIII 20,000 IU per year)	Hemophilic arthritis of both hip and knee	Bilateral THA	Hemophilic pseudotumor with large bone defects (following periprosthetic fracture)	Disarticulation of left hip, right TKA	Left hip wound unhealing, received debridement three times. The wound healed after 6 months' dressing, walking with crutches (3 years after amputation)
2	A, severe	No	17 years	Inadequate secondary prophylaxis. (irregular FVIII injection on demand)	Pathological fracture of right femur	Bone traction of right femoral fracture	Pathological fracture, internal fixation failure, hemophilic pseudotumor with infection	Above-knee amputation of right side	No complication, walking with crutches (3 years)
3	A, moderate	No	Since childhood (26 years)	Regular primary prophylaxis (FVIII approx. 5,000 IU per year)	Hemophilic arthritis with chronic osteomyelitis on right knee	Debridement of right knee ulcerative wounds	Knee stiffness with chronic osteomyelitis	Above-knee amputation of right side	No complication, walking with crutches (1 year)
4	A, moderate	No	Since childhood (24 years)	Regular primary prophylaxis (FVIII injection)	Hemophilic pseudotumor of right thigh, with systematic infection	N/A	Hemophilic pseudotumor with systematic infection	High-thigh amputation of right side	Transferred to ICU for 3 days after the operation, no postoperative complication; died from epilepsy two years after amputation

**Table 2:** Risk factors summarized from amputation patients' records and their preferences in reported cases.

Risk factor		Amputation patients no.	All patients no.	*P* value	Frequency in reported cases
Hemophilic pseudotumor	Yes	3	21	0.00	48.15% (13/27)
	No	1	180	—	—
Infection	Yes	3	10	0.00	51.85% (14/27)
	No	7	191	—	—
Factor inhibitor	Yes	1	1	N/A^*∗*^	29.63% (8/27)
	No	200	200	—	—

^∗^The statistical analysis was not performed for factor inhibitor because there was only one patient included.

**Table 3:** Previous reported amputation cases.

Case number	Hemophilia type and main concomitant diseases	Musculoskeletal involvement	Surgical procedures	Reason for amputation
1 [11]	Moderate HA	Pathological fracture with HPT on right femoral shaft	Closed reduction and external fixation of right thigh; HPT excision with open reduction and internal fixation of tight thigh; high-thigh amputation	Pin infection, internal fixation failure, HPT recurrence
2 [11]	Severe HA	Pathological fracture with HPT on left femoral shaft	High-thigh amputation	Fracture and enlargement of HPT
3 [11]	Moderate HB	Pathological fracture with HPT on left femoral shaft; HPT on right femoral shaft with secondary pathological fracture on tight femoral shaft	Disarticulation of left hip; high-thigh amputation of right side	Fracture and enlargement of HPT
4 [9]	Severe HA	HPT, fracture of right femur	External fixation for femur fracture; Removal of external fixator, HPT excision and open reduction with internal fixation; amputation of left thigh at last	Nail infection, internal fixation failure
5 [9]	Mild HA	HPT, cutaneous fistulas and infection on thigh	Amputation of right thigh	Infection
6 [9]	Mild HA	HPT, cutaneous fistulas and infection on knee	Amputation of right thigh	Infection
7 [9]	Moderate HA with inhibitor	HPT with cutaneous fistulas and infection on left thigh	Amputation of left thigh	Infection
8 [9]	Severe HB	HPT with cutaneous fistulas and infection on both thigh and left knee	Left thigh amputation; right HPT excision; right thigh amputation	Infection for left thigh, HPT recurrence with osteolysis for right thigh
9 [9]	Moderate HA	HPT, cutaneous fistulas and infection on thigh	Amputation of left thigh	Infection
10 [9]	Moderate HA, with inhibitor	HPT, cutaneous fistulas and infection on left calf	Amputation of left calf	Infection
11 [9]	Severe HA	Massive HPT on left thigh	Amputation of left thigh	Enlargement of HPT
12 [9]	Severe HA	Massive HPT on right thigh	Amputation of right thigh	Enlargement of HPT
13 [16]	Moderate HA	Huge HPT with osteolysis on right femur	Hematoma evacuation, debridement and hip hemiarthroplasty; right hip dislocation	Wound infection with ulcer, hip prosthesis dislocation
14 [16]	Mild HA	Primary benign bone giant cell tumor on right distal fibula	Intralesional cottage (2 times); aboveknee amputation	Severe hematoma and wound dehiscence
15 [17]	Severe HA	HPT with osteolysis on distal phalanx of left little finger	Left little finger amputation	Persistent infection and enlargement of HPT
16 [17]	Severe HA	HPT with osteolysis on middle phalanx of left little finger	Left little finger amputation	Persistent infection and enlargement of HPT
17 [18]	N/A	Massive HPT on knee	Intralesional excision of pseudotumor with bone cement spacer implantation; above-knee amputation	Pseudotumor recurrence after excision
18 [19]	Moderate HA	Pathological fracture, then recurrent HPT on left femur	Open reduction and intramedullary fixation and a revision surgery; left lower limb amputation	Recurrent pseudotumor and persistent swelling of left limb
19 [19]	Severe HA with low titre of inhibitor	HPT on right tibia and fibula	Right lower limb amputation	Pain and arterial ulcer
20 [20]	HA, AIDS, hepatitis B and C	Huge HPT on right femur	Right hip dislocation	Hematoma enlargement with spontaneous bleeding, heart and kidney failure
21 [21]	Severe HA with high titre of inhibitor, hepatitis C, diabetic mellitus	Huge HPT and secondary compartment syndrome on left distal limb	Supracondylar amputation of left limb	Severe compartment syndrome with phlegmon
22 [22]	Severe HA with high titre of inhibitor,	Hemophilia arthritis	Knee replacement of left side; revision of left knee; above-knee amputation of left knee	Uncontrolled infection after knee replacement
23 [23]	Severe HA	HPT with osteolysis on second metacarpal bone of right hand	Ray amputation of right index finger	Enlargement of HPT limited hand move
24 [24]	Severe HA	Multiple HPT with osteolysis on right femur, knee and tibia	Amputation of left lower limb	Enlargement of HPT and limitation of move
25 [25]	Severe HB with low titre of inhibitor	Multiple HPT with osteolysis of right proximal tibia and foot, with necrotic of left foot soft tissue	Transfemoral amputation of left lower limb	Enlargement of HPT with infection
26 [26]	Moderate HA with high titre of inhibitor	Pathological fracture followed by HPT on left tibia/fibula	Left knee disarticulation	Wound infection
27 [27]	Moderate HA	Hemophilia arthritis	Proximal tibial osteotomy of left side; Anterior and posterior fasciotomies; above-knee amputation	Deep venous thrombosis

HA, hemophilia A; HB, hemophilia B; HPT, hemophilic pseudotumor; N/A, not referred.

## Data Availability

The datasets used and/or analyzed during the current study are available from the corresponding author on reasonable request.

## Supplementary Materials

Supplementary Materials Table S1: perioperative and follow-up results for patients who received amputation.
