# Supplementary material for: Risk Factors for Amputation in the Surgical Treatment of Hemophilic Osteoarthropathy: A 20-Year Single-Center Report
Source: Pain Res Manag. 2022 Mar 28;2022:1512616. doi: 10.1155/2022/1512616 (PMC8979685; doi:10.1155/2022/1512616)
Supplement: Supplementary Materials — Table S1: perioperative and follow-up results for patients who received amputation. [file 1512616.f1.docx]

**Supplementary Material**

| **Table S1.** Perioperative and follow-up results for patients who received amputation | | |  |  |  |
| --- | --- | --- | --- | --- | --- |
| Patient | 1 | 2 | | 3 | 4 |
| Anesthesia | General anesthesia | General anesthesia | | General anesthesia | General anesthesia |
| Operation method | Dislocation of left hip and TKA of the right knee | Above-knee amputation of the right limb | | Above-knee amputation of the right limb | Amputation of right thigh at a high level with pseudotumor resection |
| Perioperative replacement agent | rhFVIIa, PCC (3 days after amputation) | hFVIII | | hFVIII | hFVIII |
| Secondary operation | - Pseudotumor resection of the left hip wound with NPWT insertion, debridement of right knee wound - Wound debridement and NPWT replacement of the left hip - Remove of NPWT in left hip, debridement, and suture of the wound in the left hip and right knee | None | | None | None (transferred to ICU for 3 days after the operation) |
| Discharge time | 63 days after amputation | 6 days after amputation | | 6 days after amputation | 21 days after amputation |
| Recent follow-up | The wound healed after 6 months’ dressing, walking with crutches (3 years after operation). | No complications. The recovered ability for self-care (3 years after operation). | | No complications. The recovered ability for self-care (1 year after operation). | No complications. Death from epilepsy two years after amputation. |
| rhFVIIa: recombinant human factor VIIa; PCC: prothrombin complex concentrate; hFVIII: human factor VIII; NPWT: negative pressure wound therapy | | |  |  |  |
